# The mediating role of social support and resilience in the relationship between social identity and mental health among international students

**DOI:** 10.1192/bjo.2025.72

**Published:** 2025-06-05

**Authors:** Melisa Parlak, Daniel Michelson, Matthew J. Easterbrook

**Affiliations:** School of Psychology, University of Sussex, UK; Department of Child and Adolescent Psychiatry, Institute of Psychiatry, Psychology and Neuroscience, King’s College London, UK; NIHR Maudsley Biomedical Research Centre, South London and Maudsley NHS Foundation Trust and King’s College London, UK

**Keywords:** Group memberships, social support, resilience, mental health, international students

## Abstract

**Background:**

International students account for a growing proportion of university students and can experience mental health challenges. While the Social Identity Model of Identity Change (SIMIC) highlights the mental health-promoting benefits of preserving and building group memberships, it overlooks the effects of identifying with a particularly salient group such as fellow international students.

**Aims:**

This study aimed to explore how SIMIC and international student identification contribute to understanding the protective factors that predict students’ mental health.

**Method:**

A cross-sectional survey of 343 international students employed path analyses to examine the associations among identification with fellow international students, social identity maintenance, new group memberships and psychological distress, with social support and resilience as potential mediators. Indirect effects were evaluated using 95% confidence intervals.

**Results:**

New group memberships (*β* = −0.01; *P* = 0.05; 95% CI = −0.03, −0) and identification with international students (*β* = −0.02; *P* = 0.05; 95% CI = −0.02, −0) predicted psychological distress, both mediated by social support and resilience. While the maintenance of previous groups did not indirectly predict psychological distress through these mechanisms (*β* = −0.01; *P* = 0.13; 95% CI = −0.02, 0), a significant indirect effect (*β* = −0.04; *P* = 0.03; 95% CI = −0.09, −0) was observed through social support when accounting for covariates.

**Conclusions:**

Shared international student identity and new group memberships offer a sense of social support and resilience which, in turn, alleviates psychological distress. Interventions could reframe this identity as a source of strength for international students.

International students, defined as individuals who cross national borders for education,^1^ numbered 6.4 million in 2023.^2^ The UK is the second most popular destination, hosting 758 855 international students in the 2022/23 academic year.^3^ While studying abroad offers significant opportunities, international students also face a range of psychosocial and academic stressors,^4^ including language barriers,^5^ social isolation^6^ and discrimination,^7^ all of which can adversely affect their mental health and may contribute to depression and anxiety.^8^

Despite rising demand, many institutions continue to fall short in addressing international students’ mental health concerns.^9^ Culturally sensitive services remain limited^10^ and available support primarily addresses immediate challenges, which has been shown to be insufficient for promoting this group’s long-term well-being.^11^ Moreover, international students often hesitate to seek professional help due to unfamiliarity with local procedures and a shortage of cross-culturally trained professionals, which suggests that their unique experiences may not be fully understood.^12^

A more integrated and effective, and potentially less stigmatising, approach to understanding international students’ experiences would involve examining the emotional and cognitive ties they share with their social groups. Evidence indicates that meaningful group memberships play a crucial role in promoting the well-being and mental health of these students.^13^ Drawing on social identification theory,^14^ which posits that meaningful group memberships are crucial for adapting to life changes,^15^ this approach could better inform support strategies tailored to the unique needs of international students.

Central to this approach is the idea that social identification provides access to resources that protect well-being and mental health during life transitions, especially through multiple group memberships.^16^ These resources include diverse support networks,^16^ a strong sense of belonging,^17^ reduced depressive symptoms^18^ and increased resilience.^19^ The benefits of belonging to multiple groups are particularly evident during significant life events and transitions.

## The Social Identity Model of Identity Change (SIMIC)

The Social Identity Model of Identity Change (SIMIC),^20^ based on social identity theory,^16^ provides a framework for navigating significant life changes − particularly those involving identity loss or threats to the self − by leveraging multiple group memberships^21^ in line with the social cure approach.^21^ This model is particularly relevant to international students, because it proposes two key pathways for supporting their adjustment and mental health. The first pathway, social identity maintenance, posits that preserving group memberships during transitions enhances social identity capital and self-continuity.^21^ However, relocation often leads to the loss of some memberships.^22^ In such cases, the social identity gain pathway facilitates adaptation by fostering new social identities.^21^

Initial support for SIMIC was found in a study of stroke survivors,^23^ and the framework has since been reinforced in regard to motherhood^24^ and students transitioning to university.^25^ Other studies have demonstrated the positive impact of SIMIC on reducing stress, depression, anxiety and loneliness.^16^

Research on international students shows valuable insights but presents mixed findings on which pathways precisely predict mental health. Quantitative studies suggest that social identity maintenance improves academic performance, while social identity gain did not predict academic or mental health outcomes among international students in Australia.^26^ Depression and life satisfaction, meanwhile, have been linked to identity loss rather than to identity gain or maintenance.^27^ Qualitative evidence suggests that factors such as host family support and culture shock can either facilitate or hinder social identity change.^22^ Additionally, our previous research indicates that challenges in establishing SIMIC pathways − such as language barriers − can exacerbate mental health issues by increasing anxiety and loneliness.^28^

## Social support, resilience and group identification

While evidence suggests that group identification enhances key psychosocial resources,^16^ other research indicates that these factors interact with each other in shaping well-being outcomes. For instance, studies showed that social support strengthened resilience which, in turn, reduced perceived stress among university students.^29^ Conversely, limited social support and thereby lower resilience were linked to greater psychological distress among refugees in Germany.^30^ These findings offer valuable insights into how social support and resilience are linked to mental health, yet more comprehensive research is needed to explain how these factors, arising from group memberships, directly associate with international students’ mental health.

We extend this literature by proposing that international students’ resilience derives from two social identity determinants: multiple group memberships − including both SIMIC pathways − and a strong, singular identification with the international student community. Evidence from diverse contexts supports this idea that meaningful group memberships bolster athletes’ recovery^19^ and enhance trauma coping when social identities are sustained or newly formed.^20^ Single group identification can also strengthen resilience, as observed among New Zealand adolescents.^31^ In our qualitative study, we found that identifying with fellow international peers − both within and outside cultural circles − boosts adjustment and well-being by fostering solidarity and resilience. Similarly, reinforcing international student identity improved self-esteem and buffered against feelings of exclusion.^32^

## Current study

While SIMIC highlights the benefits of multiple group memberships for well-being, its relevance to the mental health of international students remains ambiguous, even though qualitative evidence suggests a potential link, and the potential benefits of single, meaningful group identification have been overlooked in their association with the mental health of international students. To address these gaps, this study aims to explore how SIMIC pathways and international student identification relate to psychological distress, with social support and resilience as mediators.

**Fig. 1:**
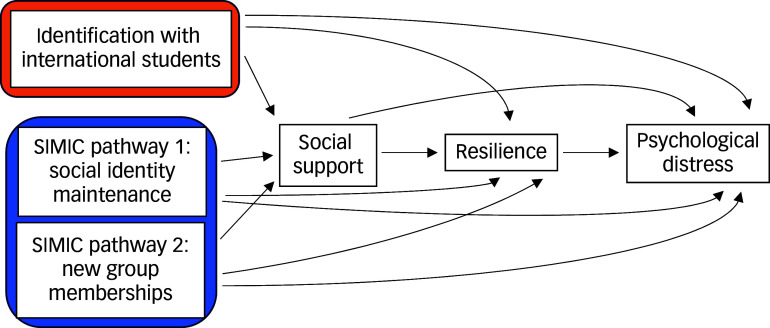
The model. SIMIC, Social Identity Model of Identity Change.

Specifically, our model (Fig. 1) fills a key gap in the literature by positing that stronger group identification − whether through multiple or a single group − reduces distress by boosting social support and resilience. We therefore hypothesise a serial mediation effect, informed by existing literature on similar populations in which greater group identification fosters social support, bolstering resilience and ultimately reducing distress, to address ambiguities in the existing literature. This integrated approach provides a nuanced understanding of how shared identities promote mental health among international students, and highlights the need for evidence-based frameworks to better support this group during major life transitions.^21^

## Method

### Design

This study is pre-registered on OSF (https://osf.io/5wgy9/?view_only=618b77ad56044f848377452b0277b47b) and utilises a cross-sectional survey design surveying international students across various UK universities. Data collection was conducted using Qualtrics from November 2023 to May 2024.

### Participants

Path modelling is recommended to include a minimum of 200 participants and approximately 10 participants per estimated parameter.^33^ With around 47 parameters, we therefore aimed to recruit between 200 and 500 participants. Due to time constraints, our final sample comprised 343 international university students enrolled at UK universities, providing adequate statistical power for path analyses. To be eligible to participate, participants had to meet specific criteria: (a) not being a UK citizen or permanent resident, (b) having legal residence outside the UK and (c) holding a student visa for educational purposes within the UK. The participants represented a diverse range of nationalities from 75 countries and were enrolled in 60 UK universities. Among participants there were 118 male and 217 female students, with ages ranging from 18 to 30 years and older (*M* = 22, s.d. = 3.27).

### Procedure

The authors assert that all procedures contributing to this work comply with the ethical standards of the relevant national and institutional committees on human experimentation, and with the Helsinki Declaration of 1975 as revised in 2013. All procedures involving human subjects/patients were approved by the relevant ethnics committee at the university of the first author (ER/MP668/4). We adopted volunteer sampling via flyers, social media and campus support services. For rapid recruitment of eligible participants, we supplemented these efforts with convenience and snowball sampling through the first author’s network.

Promotion included distributing flyers with contact details and a QR code throughout the institution, as well as outreach to university support services and cultural societies via email to encourage international student participation. The survey link was shared on various social media platforms, including Facebook, WhatsApp, Reddit and Discord channels related to UK universities. Additionally, the third author facilitated the survey’s implementation on the SONA system, offering Psychology undergraduate students course credits for completion.

Prospective participants accessed the survey via a unique code from flyers or through a shared link. Following entry, they underwent an initial screening to verify their eligibility as international students. Eligible participants reviewed the participant information sheet and provided electronic written informed consent. They were also given the option to enter a prize draw by submitting their email addresses. Participants then completed a series of questionnaires assessing their experiences with previous and new groups, identification with fellow international students, resilience, perceived social support and psychological distress. The survey took approximately 10 min to complete.

### Measures

Demographics. After obtaining informed consent, participants provided personal information, including gender, age, home country, first language, additional languages spoken and prior relocation experience. They also shared their university affiliation, field and level of study, type of accommodation, duration of stay in the UK (in years) and self-assessed English language proficiency (*α* = 0.92).^34^

Attention checks. The survey included two attention check questions, instructing participants to respond with ’strongly disagree’ and ‘agree’, respectively. Participants who answered both attention checks incorrectly were excluded from the analysis, resulting in the removal of 57 individuals.

Maintained group memberships. A 4-item Maintained Group Memberships scale^23^ measured the degree to which participants were able to maintain their pre-existing social group membership during the transition, on a 7-point Likert-type scale from ‘do not agree at all’ (1) to ‘agree completely’ (7) (*α* = 0.80). Under the prompt ‘After moving to the UK’, one of the example items included: ‘I continue to maintain strong ties with the same groups as before moving to the UK’. Higher scores suggest the ability to maintain previous group memberships. Therefore, this scale captures how continuity in pre-existing social identities may buffer stress during transition.

New group memberships. A 4-item New Group Memberships scale^23^ measured participants’ strength of association with new social groups following their life transition, on a 7-point Likert-type scale from ‘do not agree at all’ (1) to ‘agree completely’ (7) (*α* = 0.90). Under the prompt ‘After moving to the UK’, example items included: ‘I have joined the activities of new groups’. Higher scores indicate a greater ability to form new group memberships, a construct that assesses the formation of new social ties which, in turn, may facilitate integration and enhance mental health.

Identification with international students. The extent of international students’ identification with their international peers was explored using the 10-item In-Group Identification Measure,^35^ on a 7-point Likert-type scale from ’strongly disagree’ (1) to ’strongly agree’ (7). This measure assesses the degree to which international students feel connected, content and perceive themselves as integral members of the international student community (*α* = 0.83). Participants rated items such as ‘I feel committed to other international students’, with higher scores reflecting greater identification.

Perceived social support. Participants responded to a 4-item Social Support Scale that was used to measure perceived social support on a 7-point Likert-type scale,^36^ ranging from not at all (1) to completely (7) (*α* = 0.91), with higher scores reflecting greater perceived social support. For example, one item asked: ‘Do you get the emotional support you need from other people?’.

Resilience. A 10-item Connor–Davidson Resilience scale^37^ measured students’ resilience on a 5-point Likert-type scale, from ‘not true at all’ (0) to ‘true nearly all the time’ (4) (*α* = 0.88). Among these items were: ‘I believe I can achieve my goals, even if there are obstacles’ and ‘I am able to adapt when changes occur’. Higher scores reflect greater resilience, indicating an individual’s capacity to effectively cope with stress and adapt to challenges.

Mental health. The study utilised the 12-item version of the General Health Questionnaire (GHQ-12)^38^ to gauge psychological distress effectively. This questionnaire is widely recognised for its utility in identifying mental health issues across student samples.^39^ We used the Likert method (0, 1, 2, 3), providing a potential score range from 0 to 36 (*α* = 0.71). For instance, one item asks, ‘Have you recently been thinking of yourself as a worthless person?’, with higher scores indicating greater psychological distress.

### Data analysis

Hypotheses were tested using path analyses in statistical software R version 4.3.3 for Windows 11 Pro (R Foundation for Statistical Computing, Vienna, Austria; see https://cran.r-project.org/), with the lavaan package version 0.6-17 (Yves Rosseel, Ghent University; see https://lavaan.ugent.be/), by the first author. Missing data were handled using Full Information Maximum Likelihood. Predictor variables included identification with international students, social identity maintenance and new group memberships, while the outcome variable was psychological distress. Perceived social support and resilience were included as mediator variables in the analyses. To assess the robustness of our path mediation results, we controlled for the following covariates: age, gender, English language proficiency, number of languages spoken, length of stay in the UK and prior relocation experience.

## Results

### Sample characteristics

The participants were 343 international students, aged 18–30 years, who were enrolled at universities across the UK. The majority were female (*n* = 217, 64.8%) and Indian (*n = 41,* 12%). The mean age of participants was 22.0 years (s.d. = *3.27).* Overall, students represented 60 different UK universities and came from 75 countries.

Of the participants the majority were undergraduates (*n* = 207, 61.24%), including 94 first-year undergraduates (27.5%). The sample also included 96 Master’s (28.4%) students and 35 Doctoral (10.36%) students. Participants had lived in the UK for an average of 1.06 years (s.d. = 1.26). Most participants lived in privately rented accommodation (*n* = 165, 48.4%), followed closely by those in university accommodation (*n* = 163, 47.8%), while a small minority resided in a family home (*n* = 13, 3.8%). The correlations and descriptives of study variables are presented in Table 1.

**Table 1:** Descriptive statistics and correlations for study variables

Variable	*N*	Mean (s.d.)	Range	Bivariate correlations					
				1	2	3	4	5	6
1. Resilience	339	27.6 (6.86)	0–40	1					
2. Social support	337	16.3 (5.52)	4–28	0.26	1				
3. Psychological distress	328	15.2 (7.68)	0–36	−0.39	−0.30	1			
4. Identification with international students	335	49.6 (9.76)	10–70	0.27	0.36	−0.18	1		
5. New group memberships	333	20.8 (5.45)	4–28	0.24	0.34	−0.16	0.28	1	
6. Maintenance of groups	340	18.5 (5.12)	4–28	0.07	0.18	−0.08	0.24	0.05	1

### Test of the model

Path analyses were conducted to assess how perceived social support and resilience mediate the relationships among identification with international students, social identity maintenance, new group memberships and psychological distress. Table 2 displays the required values for the indirect effects, including effect sizes, *P*-values and 95% confidence intervals.

**Table 2:** Mediation paths with indirect effects on psychological distress

Mediation pathways	Predictor	Outcome	*B* (*β*)	s.e.	*Z*-value	*P*-value	95% CI lower	95% CI upper
1. Perceived social support as a mediator								
1.1.	Identification with international students	Psychological distress	−0.04*	0.02	−2.94	0	−0.07	−0.01
1.2.	New group memberships	Psychological distress	−0.08*	0.02	−2.96	0	−0.12	−0.02
1.3.	Social identity maintenance	Psychological distress	−0.03	0.02	−1.81	0.07	−0.07	0
2. Resilience as a mediator								
2.1.	Identification with international students	Psychological distress	−0.05*	0.02	−2.79	0	−0.08	−0.01
2.2.	New group memberships	Psychological distress	−0.07*	0.03	−2.35	0.02	−0.12	−0.01
2.3.	Social identity maintenance	Psychological distress	0.01	0.03	0.23	0.82	−0.05	0.06
3. Perceived social support and resilience as sequential mediators								
3.1.	Identification with international students	Psychological distress	−0.01*	0	−1.98	0.05	−0.02	−0
3.2.	New group memberships	Psychological distress	−0.02*	0.01	−1.98	0.05	−0.03	−0
3.3.	Social identity maintenance	Psychological distress	−0.01	0	−1.50	0.13	−0.02	0

*Significant at *P* < 0.05 level.

**Fig. 2:**
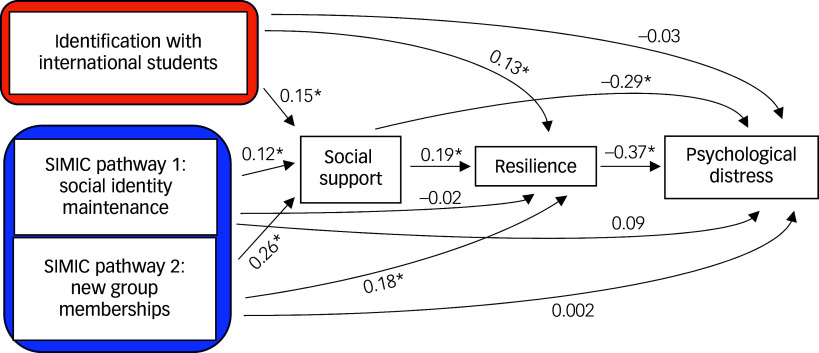
Direct effects. SIMIC, Social Identity Model of Identity Change; *P < 0.05.

As shown in Fig. 2, social support was positively predicted by identification with international students, new group memberships and social identity maintenance. Resilience, in turn, was positively predicted by perceived social support, identification with international students and new group memberships, but not by social identity maintenance (*P* = 0.82, 95% CI = −0.16, 0.13). Examining the indirect effects through social support reveals that resilience was predicted by identification with international students and by new group memberships, both via social support. There was not a significant indirect effect from social identity maintenance through social support to resilience (*P* = 0.10, 95% CI = −0, 0.05).

Psychological distress was negatively associated with resilience and perceived social support, though not directly with identification with international students, new group memberships or social identity maintenance (all *P* > 0.05). However, identification with international students and new group memberships was linked to reduced distress through two mechanisms: (a) increased social support and (b) enhanced resilience. Additionally, identification with international students and new group memberships indirectly predicted lower distress via a combined pathway of social support and resilience. No significant associations were found between social identity maintenance and psychological distress through either social support or resilience, alone or combined (all *P* > 0.05)

Analysis of covariates. Adding the covariates to the model revealed that gender (*β* = 2.48, *P* = 0.001, 95% CI = 0.99, 3.98) and perceived English language proficiency (*β* = 0.61, *P* = 0.001, 95% CI = 0.26, 0.96) significantly predicted resilience, with males and those with higher proficiency reporting greater resilience. Conversely, a greater number of languages spoken was associated with lower perceived social support (*β* = −0.95, *P* = 0.01, 95% CI = −1.68, −0.22), while a longer stay in the UK was linked to higher psychological distress (*β* = 1.34, *P* < 0.001, 95% CI = 0.60, 1.68).

The previously significant indirect effect of new group memberships on resilience became marginally significant (*β* = 0.14, *P* = 0.056, 95% CI = −0, 0.28). Additionally, the previously non-significant relationship between social identity maintenance pathway and psychological distress became marginally significant (*β* = 0.16, *P* = 0.054, 95% CI = −0, 0.32), suggesting that greater maintenance of old groups was linked to increased psychological distress in the UK.

Regarding indirect effects, the previously significant indirect effect of new groups on psychological distress via resilience became statistically non-significant (*β* = −0.05, *P* = 0.07, 95% CI = −0.10, 0), while the effect via social support and resilience became marginally significant (*β* = −0.02, *P* = 0.053, 95% CI = −0.03, 0). Moreover, the total indirect effect of new group membership became statistically non-significant (*β* = −0.08, *P* = 0.06, 95% CI = −0.16, 0). Surprisingly, a new indirect effect of identity maintenance on psychological distress via social support emerged (*β* = −0.04, *P* = 0.03, 95% CI = −0.09, −0).

## Discussion

This cross-sectional study is the first to integrate the SIMIC model with single group identification to examine their association with psychological distress through protective mechanisms for international students. The results revealed that maintaining previous group memberships, forming new ones and identifying with international students all positively and uniquely predicted social support which, in turn, positively predicted resilience, which itself negatively predicted psychological distress.

Consistent with SIMIC, forming new group memberships was associated with lower psychological distress via heightened social support and resilience. Importantly, our findings reveal that identification with fellow international students also predicted reduced distress through these mediators. However, contrary to the SIMIC consensus, maintaining group memberships did not show the expected benefits. These findings not only highlight the importance of new group memberships but also extend the SIMIC framework by revealing the crucial role of the international student identity.

The results are aligned with existing research, emphasising the protective role of group memberships to international students’ well-being,^26–28^ while also suggesting that social support and resilience serve as key mechanisms for enhanced mental health.^29^ Additionally, the findings reinforce both the theoretical and empirical foundations of the social identity approach to health,^40^ particularly highlighting the positive impact of new group memberships during significant life transitions. While SIMIC explains how group memberships protect individuals during life transitions, it does not focus on the strength of identification with specific, highly relevant social groups. Our study addresses this gap by focusing on the international student social identity, showing that identification with this group is linked to lower psychological distress through enhanced social support and resilience. This finding underscores the importance of the international student identity in mitigating mental health issues.

Despite evidence emphasising the importance of maintaining prior identities during life transitions,^23,26^ we found that maintaining previous group memberships did not predict psychological distress through social support or resilience, nor did it directly predict either resilience or psychological distress. Surprisingly, these results align with research suggesting that connections to previous groups may hinder successful integration for international students.^41^ Instead, joining new groups within the host culture has been shown to promote adaptation, enhance self-esteem and reduce distress.^41^ Additionally, resilience is more strongly associated with support from within the host country, while support from home country groups is less positively linked.^41^

Even after controlling for covariates, most effects remained significant or approached significance. Notably, the previously non-significant association of maintaining old group memberships on psychological distress became marginally significant, suggesting that holding onto previous identities may increase distress. This supports our earlier interpretation that maintaining previous group memberships can impede successful integration and increase distress among international students.^41^ Additionally, an indirect effect of maintaining group memberships on psychological distress emerged through social support, although this effect was not further mediated by resilience. Overall, the protective mechanisms of forming new group memberships and maintaining previous ones may operate differently for international students, with a tendency to favour new group memberships.

Individuals who perceived their English language proficiency as being higher reported greater resilience, probably due to their enhanced ability to navigate daily life and form new groups.^42^ However, multilingual students reported lower levels of perceived social support, possibly because navigating diverse linguistic and cultural contexts can hinder their ability to establish meaningful connections. Ultimately, the perception of support as either comforting or distressing depends on recognising a shared identity within groups,^36^ a recognition that could be influenced by their multilingualism. Nevertheless, their ability to switch between languages and cultural frameworks may lead to a fragmented sense of social identity, reducing their perceptions of support.

In summary, this study demonstrates that shared identities are essential for providing social support and resilience which, in turn, predict lower psychological distress. Our findings align with the social cure approach and SIMIC’s view that acquiring new group memberships is particularly effective in lowering psychological distress through protective mechanisms for international students, whereas maintaining pre-existing group memberships does not seem to offer similar mental health benefits to this population. Additionally, we offer new insights into the identity of being an international student and its benefits for mental health. These insights provide researchers with a new direction for developing interventions to better support international students during their transition to new environments.

### Strengths, limitations and future directions

Because this study employed a cross-sectional correlational design, it cannot test causal relationships. We therefore cannot determine whether maintaining positive mental health leads to stronger new group formation, stronger identification with international students or maintained social support, or whether these factors are consequences of experiencing less psychological distress. Although evidence in the literature supports the predictive role of SIMIC processes against mental health challenges,^21^ future research should utilise longitudinal or experimental data to examine these associations and explore the mechanisms within a causal framework.

This study examined the association between identification with international students and mental health through its protective mechanisms. International students often share similar cultural backgrounds and challenges, which foster strong bonds and mutual support during the early stages of adjusting to a new environment. In contrast, identification with home students – although potentially advantageous as posited by the Contact Hypothesis,^43^ which argues that intergroup contact can reduce prejudice and improve relationships – may be limited by language barriers and cultural differences, thereby reducing its immediate supportive impact. Consequently, our research focuses on international student identification to investigate how shared identities are associated with mental health. Future studies should consider exploring home student relationships to further clarify these protective mechanisms.

Additionally, the study’s design shows further limitations. This is partly due to our reliance on self-reported surveys completed online, with varying completion times among participants. Furthermore, the research was conducted over an academic term, predominantly involving first-year undergraduate students. It is possible that those surveyed early in their studies faced more significant challenges in group formation and adjustment to life abroad, leading to higher psychological distress. In contrast, students completing the survey later in the spring may have had more opportunities to establish meaningful identities, potentially mitigating some initial difficulties.

Moreover, we did not address other subpopulations of international students, such as refugees and asylum seekers, because our screening focused only on those with student visas. We also did not enquire whether participants came to the UK involuntarily, such as through family pressure. Future research should further investigate SIMIC in relation to these groups, through quantitative or qualitative studies. Despite these limitations, our study included 343 students from 75 countries across 60 universities in the UK, providing a diverse sample that captures a broad range of perspectives and responses from international students. This diversity enhances the relevance of our findings and lays a foundation for future research in this area.

### Implications

Building on existing literature and our recent qualitative study,^28^ our findings enhance understanding of how to support international university students. Specifically, we found that resilience from new group memberships, particularly identification with fellow international students, predicts reduced psychological distress.

While our research indicates that identification with international students can be beneficial, previous work has highlighted that this identity can also carry stigma.^32^ Recent studies^10^ also reveal that international students are frequently unfairly blamed for their difficulties. However, our findings from a prior qualitative study^28^ suggest that, when framed positively, the international student identity can serve as a source of strength. Thus, identity-reframing interventions^44^ that emphasise the unique strengths inherent in their identity – rather than focusing on challenges – could significantly enhance their well-being and mental health.

Specifically, identity-reframing interventions emphasise the strengths of individuals from low-status groups, reshaping both self-perceptions and societal views.^44^ By challenging dominant narratives that portray low-status group members as deficient, this intervention has improved goal achievement among refugee students.^44^ Inspired by these outcomes, we aim to adapt this intervention for international students, focusing on how reframing their identity can enhance their well-being and other outcomes. This aligns with the growing recognition of social determinants of health^16^ and the importance of social identity-based interventions in promoting the well-being of international students.

Although these interventions show promising benefits, their impact may remain limited without institutional support. Higher education institutions need to hire culturally diverse staff who can integrate these strategies across both counselling and academic departments. This approach would empower international students by addressing their academic and well-being outcomes more cohesively.^10^ In fact, research indicates that support services should prioritise building international students’ resilience rather than merely tackling their immediate challenges.^10^ By emphasising students’ strengths and resilience, identity-reframing interventions can enhance integration, improve mental health and foster a stronger sense of solidarity.

While universities offer support beyond academics – through student unions and cultural clubs celebrating cultural identities – these efforts may not fully meet international students’ needs. Our findings suggest that tailored activities designed to reinforce international student identity across diverse cultures, along with initiatives that foster connections with home students, could significantly enhance overall support.

## Data Availability

The data associated with this study are available from the corresponding author upon reasonable request.
